# Systems Biology, Neuroimaging, Neuropsychology, Neuroconnectivity and Traumatic Brain Injury

**DOI:** 10.3389/fnsys.2016.00055

**Published:** 2016-08-09

**Authors:** Erin D. Bigler

**Affiliations:** Department of Psychology, Neuroscience Center, Brigham Young UniversityProvo, UT, USA

**Keywords:** traumatic brain injury (TBI), neuroimaging, computed tomography (CT), magnetic resonance imaging (MRI), systems biology, connectivity, neuropsychology, quantitative image analysis

## Abstract

The patient who sustains a traumatic brain injury (TBI) typically undergoes neuroimaging studies, usually in the form of computed tomography (CT) and magnetic resonance imaging (MRI). In most cases the neuroimaging findings are clinically assessed with descriptive statements that provide qualitative information about the presence/absence of visually identifiable abnormalities; though little if any of the potential information in a scan is analyzed in any quantitative manner, except in research settings. Fortunately, major advances have been made, especially during the last decade, in regards to image quantification techniques, especially those that involve automated image analysis methods. This review argues that a systems biology approach to understanding quantitative neuroimaging findings in TBI provides an appropriate framework for better utilizing the information derived from quantitative neuroimaging and its relation with neuropsychological outcome. Different image analysis methods are reviewed in an attempt to integrate quantitative neuroimaging methods with neuropsychological outcome measures and to illustrate how different neuroimaging techniques tap different aspects of TBI-related neuropathology. Likewise, how different neuropathologies may relate to neuropsychological outcome is explored by examining how damage influences brain connectivity and neural networks. Emphasis is placed on the dynamic changes that occur following TBI and how best to capture those pathologies via different neuroimaging methods. However, traditional clinical neuropsychological techniques are not well suited for interpretation based on contemporary and advanced neuroimaging methods and network analyses. Significant improvements need to be made in the cognitive and behavioral assessment of the brain injured individual to better interface with advances in neuroimaging-based network analyses. By viewing both neuroimaging and neuropsychological processes within a systems biology perspective could represent a significant advancement for the field.

The *International and Interagency Initiative toward Common Data Elements (CDE) for Research on Traumatic Brain Injury (TBI) and Psychological Health* (see Menon et al., 2010) defines TBI as “… as an alteration in brain function, or other evidence of brain pathology, caused by an external force (p. 1637)” where severity is most commonly characterized by whether there was loss of consciousness (LOC) including its duration, post-traumatic amnesia (PTA) and/or Glasgow Coma Scale (GCS) ratings. While these features of TBI are important descriptors of the injury they provide only limited information about underlying neuropathology, or how the injury may relate to outcome but often, are the only uniform descriptors of a brain injury used clinically or in research, especially in neuropsychological outcome studies. The problem with this approach is immediately grasped by viewing Figure 1. Patients with identical GCS scores, or whether LOC occurred or not, may have similar or widely diverse neuropathological findings on magnetic resonance imaging (MRI) at the same chronic stage post-injury. If a neuropsychological outcome study were to use only GCS, PTA, LOC or some similar injury severity rating, cases like in Figure 1 become lumped together with incredibly diverse underlying neuropathology. This diversity of pathology also means that any singular neuroimaging metric used to assess pathology will underestimate the totality of pathological effects or fail to even detect presence of a pathological change in the brain brought on by the trauma.

**Figure 1:**
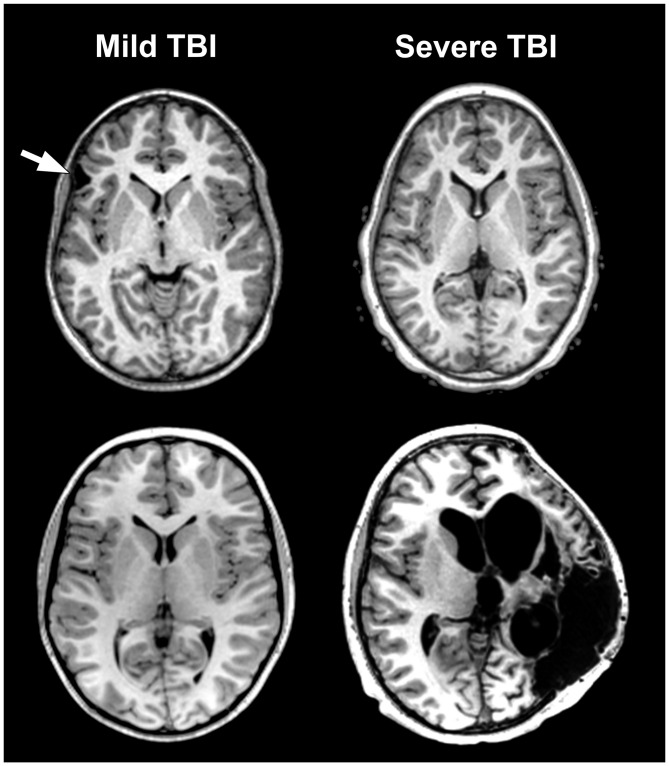
**The problem of traumatic brain injury (TBI) severity classification by using the Glasgow Coma Scale (GCS) is the wide disparity of structural pathology that may be present for a given classification level.** Traditionally, mild TBI (mTBI) has been classified by GCS scores between 13–15. The arrow in the case presented in the upper left depicts a prominent focal area of frontal encephalomalacia as a residual from an old contusion in this individual who had a mTBI and an initial GCS = 14. Note that the asymmetry of the anterior horn of the lateral ventricular system on the side of the lesion that is likely a subtle reflection of greater parenchymal volume loss surrounding the side of the lesion. In contrast, the case in the lower left or the one in the upper right have no visible abnormality, despite GCS scores of 15 and 3, respectively. Finally, the obvious massive structural damage in the lower right is from a TBI patient with severe TBI and GCS of 3. The case in the upper left had a reported brief loss of consciousness (LOC) but the other individual with mTBI (lower left) did not. Both severe TBI cases also had positive LOC.

The basis for much of the confusion generated in the neuropsychological literature about TBI outcome is likely the result of combining cases with differing TBI-related pathology examined only with basic neuroimaging metrics. For example, in Figure 1 the axial images from a MR scan of two individuals who sustained severe TBI are shown on the right side of the figure. One demonstrates no observable gross pathology while profound abnormalities are distinctly visible in the other. In the child with extensive structural pathology there is parenchymal loss, shape distortion and multiple variations in MR signal intensity that deviate from the norm, each indicating differences in the types of neuropathological changes that have occurred. For the two cases with mild TBI (mTBI) shown on the left of the figure, one had a sizeable frontal lesion, the other no abnormality, just like one of the severe TBI cases (upper right). Also evident from viewing Figure 1 is that there is a tremendous amount of information in those images about the size, volume, shape, length, thickness, etc., of brain structures, as well as visible pathology when present, all of which can be quantified. Improved identification and quantification of brain images, including a multi-modality approach to comprehensively identify abnormalities should improve the predictive ability of neuropsychological outcome studies and likewise better inform treatment and follow-up for the TBI patient. However, what neuroimaging measures to use and within what framework TBI neuropathology is identified represent complex, unresolved issues and the basis for this review.

Masel and DeWitt (2010) argue that TBI should not be viewed as an event, but as a disease process (see also, Masel, 2015). This makes sense because even though TBI clearly has an exogenously defined onset, as stated in the definition above, the injury sets into motion a cascade of various pathological effects (Johnson et al., 2013, 2015; Smith et al., 2013; Armstrong et al., 2015, 2016; Mierzwa et al., 2015), some of which may be purely short-lived and transient, while others are chronic. Chronic effects from TBI are sufficiently common and disabling that TBI meets criteria as a disorder with a major world-wide disease burden (Olesen and Leonardi, 2003). Since there is a time-dependent staging to injury effects, neuroimaging analyses need to be dynamic (Kim and Gean, 2011). If there are a multitude of pathological factors initiated by the injury, then characterizing them by various features extracted from neuroimaging variables should not be singular but as comprehensive and thorough as possible. As pointed out in Figure 1, it is a mistake to just characterize TBI by one of the markers of injury severity. It would be equally a mistake to characterize the neuroimaging identified neuropathology by a single measure (i.e., presence/absence of a focal lesion). But how should neuroimaging findings be analyzed, within what theoretical framework and how should these metrics be applied to outcome research and clinical use? What are some of the best ways to conceptualize traumatically induced neuropathology using current neuroimaging technology? These are the issues of this review.

Returning to Figure 1 the abnormalities that are highlighted reflect differences between each patient and likely relate to different aspects of TBI pathology. Given this striking heterogeneity, it immediately becomes apparent that there is no universally occurring “lesion” in TBI. It would also be unsatisfactory to approach this within a simple framework of the size or just where a definable abnormality may be located, which up to this time has been a common approach to neuropsychological outcome studies. Additionally, as will be explained more fully below, the information contained within a MR scan is unique to that individual, but most TBI studies approach neuroimaging analyses via group data comparisons. Whatever neuroimaging analysis tools emerge, they must be able to account for individual differences in brain structure and function but also appropriately identify all types of pathology potentially discernable from an image.

A systems biology framework for understanding neuroimaging findings and their relevance to neuropsychology seems a most appropriate next step to improve understanding of the effects of TBI. Adapted from Vodovotz and An (2015), Figure 2 depicts a common “systems” approach applied to any disease or disorder. Such an approach emphasizes tissue, organ and systemic levels of an integrated system influencing health and dysfunction. As depicted in Figure 2, neuroimaging can inform every level of the system, but to understand the significance of the neuroimaging finding at each level within the system, one needs to know how neuropathological changes are manifested in scan findings and whether such findings influence behavior, emotion and/or cognition. So added to the schematic offered by Vodovotz and An (2015) is the potential value of neuroimaging which appears to be well equipped to address these three levels of a systems approach. When neuroimaging is combined with neuropsychological techniques, it would seem to be a recipe for a more comprehensive explanation of TBI outcome. Of course, the “systems” approach presented in Figure 2 begins at the tissue level that includes the cellular, metabolic and molecular and it is at these levels where the TBI story begins. What can neuroimaging inform about cellular pathology when the conventional MRI standard of image acquisition is based on an inferred slab of tissue that is a cubic millimeter thick?

**Figure 2:**
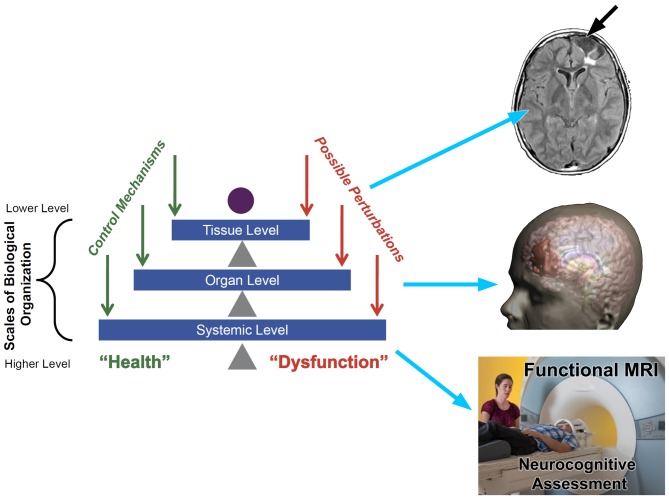
**Taken from Vodovotz and An (2015) the left side illustrates a pyramid view of a traditional systems biology approach from the tissue to systemic level where to the right, potential neuroimaging approaches to assess each level within the system is shown.** Tissue Level: upper right depicts a fluid attenuated inversion recovery (FLAIR) sequence that shows tissue loss (encephalomalacia—white arrow) and underlying region of damaged white matter (WM; bright white region). Organ Level: whole brain is depicted in the middle as a 3-D reconstruction of the head and brain where the region in red depicts the overall extent of focal encephalomalacia that was shown at the tissue level. Systemic level: whole person being behaviorally and cognitively assessed using neurocognitive assessment including functional magnetic resonance imaging (fMRI) methods while in the scanner. The “systems” illustration on the left is taken from An et al. (2012) used with permission from e-Century Publishing.

## The Injury and the Biomechanics of Trauma

TBI begins with the event that induces the injury and therefore mechanism of injury becomes a critical variable. What is quite astonishing is that even with the most precise experimental controls applied to animal models of TBI, where the identical weight-drop, fluid percussion, blast or other experimental condition is imposed the injury and resulting histology is never identically replicated (Statler et al., 2008). If the injury cannot be precisely replicated under strict experimental control, the diversity of circumstances and mechanical forces that lead to TBI in humans means that no two brain injuries are ever alike! Now add to this the fact that each brain develops within its own unique experience dependent world under unique genetic, environmental, nutritional, emotional and socioeconomic forces, no two brains are ever alike. In fact, several studies (see Finn et al., 2015; Ueda et al., 2015) have demonstrated that imaging findings are so individualized by distinctive differences in brain morphology that each brain has its own “neural fingerprint.”

The recent study by Bigler et al. (2016) is an example of the uniqueness of focal brain injuries and mechanism of injury within a large study of 251 pediatric cases where TBI was assessed, focused on identifying cases of mTBI (GCS ≥ 13). All patients were assessed within an emergency department (ED) soon after the injury. In those meeting criteria for having sustained a TBI, there were over 30 different categories related to mechanisms of injury (falls, sports related, motor vehicle, etc.) and when visible MR pathology was identified, there were no two children with pathology that was similar in size, location, distribution or identically overlapped. Also, when pathology was identified, it varied depending on the MR sequence used. All of this underscores the uniqueness of each TBI for each individual who sustains a brain injury. More to be written about later in the review.

Much of the pathology from trauma occurs as the result of tissue deformation that involves strain-related responses of the brain to impact dynamics influenced by the shape of the brain and its relation to the skull, meninges and vasculature (Bigler, 2007). The degree of deformation is influenced by acceleration-deceleration forces where the magnitude and directionality of change predict where the greatest shear-strain forces occur (Zhao et al., 2016). The biomechanical events associated with traumatic injury place tensile strain on axons which depending on the location and amount of those forces, neural tissue becomes deformed beyond biological tolerance resulting in axon damage and other ultrastructure pathology (Cloots et al., 2013; Wright et al., 2013; Sullivan et al., 2015). While true shear lesions occur (Peerless and Rewcastle, 1967), the term traumatic axonal injury or TAI may better characterize much of the microstructural damage because it includes a combination of pathological factors (Bigler and Maxwell, 2012).

To highlight the different sensitivities of MR technology in studying TBI, a case study will be used to illustrate what information can be extracted from a scan, some methods for image analysis and how different scan parameters lead to detecting different aspects of pathology. Another advantage for using a single case study opposed to group analyses is that there will be no need to provide additional demographic and injury details for a single subject, which would be required in a group analysis. Returning to what is outlined in Figure 2, the case study selected for an in-depth review should reflect the different levels of targeted information that a systems biology approach could identify to elucidate the effects of TBI. By using a case study approach, specific neuroimaging details about pathological identification that are unique to the individual can be extracted that otherwise would be lost in group data comparisons. Nonetheless, each of the points discussed in the identification of neuroimaging based pathology in this case study approach provides the basis for further empirical investigation at a group level. In-depth review of neuroimaging findings in TBI are covered in the original text by Gean (1994) titled *Imaging of Head Trauma* and recently updated (see Gean, 2015).

The child participant selected and highlighted as the case study in this review was from the Social Outcomes of Brain Injury in Kids (SOBIK) investigation, details of which have been previously published (Bigler et al., 2013; Dennis et al., 2013; Yeates et al., 2013). Hereafter, this will be referred to as the “TBI Case Study”. All aspects of the study had institutional review board approval and followed all ethical guidelines for human neuroimaging and neuropsychological studies. MRI findings reported herein were obtained from a 1.5 Tesla General Electric Signa Excite scanner using the following image sequences and parameters: sagittal acquisition 1.2 mm thick T1 FSPGR IR (repetition time (TR): 3.86 ms, echo time (TE): 1.47 ms); 5.0 mm thick dual echo proton density/T2 acquired in the axial plane (PD settings at 2800.00 ms for TR and 30.00 ms for TE); T2 settings at 2800.0 ms for TR and 90.0 ms for TE; Fluid attenuated inversion recovery (FLAIR) sequence was 3.0 mm thick (TR: 10002.00 ms, TE 111.89 ms) and the gradient recalled echo (GRE) sequence was also 3.0 mm thick with a TR: 567.0 ms with a TE of 15.0 ms. The SOBIK study was in part classroom based and therefore children within the study had to have sufficiently recovered from their TBI to be mainstreamed into some level of regular classroom placement. When scanned and tested at 12 years 4 months of age this male child obtained the following scores on the Wechsler Abbreviated Scale of Intelligence (WASI): full scale standard score of 109 (WASI Vocabulary *T*-score = 51, WASI Matrix Reasoning *T*-score = 59) with a Wechsler Intelligence Scale for Children—IV Edition Processing speed Index (PSI) score of 94. The child was originally injured when 8.5 years of age.

**Figure 3:**
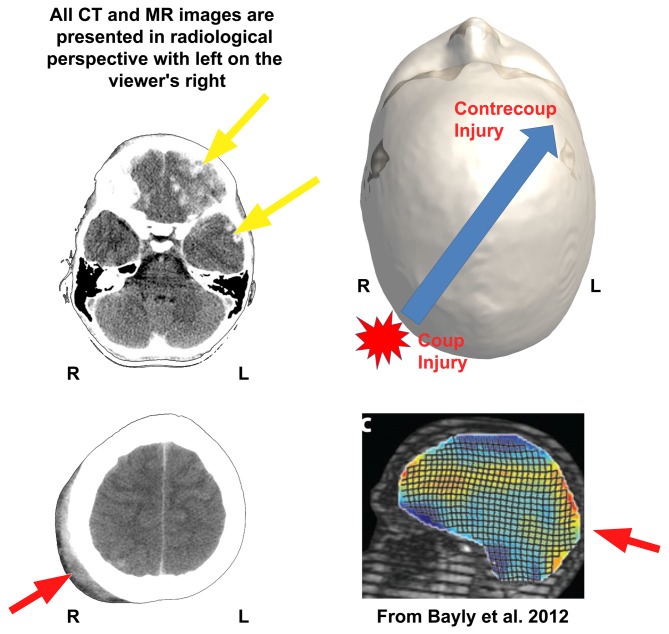
**The shear-strain effects of impact head injury are shown in the case study presented in this review.** The day-of-injury (DOI) computed tomography (CT) of this child when originally assessed in the emergency department (ED) shows the initial impact—coup injury with soft-tissue swelling outside the skull (red arrow, bottom left or red star in upper right)—influences the direction of greatest biomechanical deformation of brain parenchyma but it occurs opposite the point of impact as the contrecoup injury in the left frontal region (top yellow arrow). The red arrow in the bottom left CT shows soft tissue swelling of the scalp over the posterior right skull signifying the general area of head impact. (Note that the CT images on the left are in radiological perspective). Some of the energy of that impact becomes translated forward and opposite (contrecoup) resulting in multiple areas of surface contusion and hemorrhagic shear injury in the left frontal (top yellow arrow) and temporal lobe regions (bottom yellow arrow in the upper left CT). Although there is subtle hemorrhage (white arrow, lower left image) associated with the coup injury, the greatest amount of damage occurs secondary to the contrecoup injury (yellow arrow), where there is extensive hemorrhagic contusion and shearing involving the inferior left frontal and temporal areas. These regions of damage over time result in extensive encephalomalacia and degenerative changes as depicted in Figures 4, 6–8. The upper right image is a dorsal view of a 3-D recreation of this child’s head showing the point of impact and direction of the brain displacement resulting in the contrecoup injury (to be consistent with the radiological perspective of CT presentation on the left, please note that in the 3-D reconstruction of the head in this Figure, left is on the viewer’s right). Reproduced with permission from Bayly et al. (2012) in the lower right corner is a biomechanical demonstration of brain deformation that occurs with a head drop of 2 cm. Note that the two points of greatest deformation in a coup—contrecoup pattern. Stain magnitudes are color coded, with maximal (principal) strains shown in red. R, right; L, left.

Clinically, computed tomography (CT) is typically the first scan done when someone is acutely injured and meeting criteria for neuroimaging. Figure 3 is the day-of-injury (DOI) CT scan in the TBI Case Study child who had sustained a severe TBI (GCS = 3) as the result of a motor vehicle accident. DOI imaging is always minutes to hours after the initial injury so clinical neuroimaging never captures the immediate effects of the trauma. In this child, based on available information, the initial CT was performed at least an hour after injury, where at the scene as well as ED, GCS was rated a three. As depicted in Figure 3, CT findings demonstrated scattered hemorrhagic shear lesions and contusions. In the multisite NIH sponsored “Transforming Research and Clinical Knowledge in TBI (TRACK-TBI)” study intraparenchymal shear injuries as viewed in Figure 3 were classified as “hemorrhagic axonal injury” (see Yuh et al., 2013). Also, within the TRACK-TBI study, TAI and diffuse axonal injury (DAI) were defined by NIH CDE criteria where 1 to 3 foci were designated as TAI and ≥4 foci as DAI (Duhaime et al., 2010). These lesions are thought to occur as a consequence of shear forces deforming parenchyma and blood vessels sufficient to mechanically disrupt and/or tear tissues and the vasculature, along with surface contusions against the boney cranial fossa (Bigler, 2007). Importantly, note in the two axial views, in radiological perspective with the patient’s right side is on the viewer’s left, that the soft tissue swelling (see red arrow), where initial impact occurred, is on the right. This swelling is in the contralateral posterior aspect of the other hemisphere, opposite to where the greatest amount of traumatic shear/contusion injury occurred in the inferior left frontal region. This represents a classic coup (right parietal, initial impact) with greatest injury in a contrecoup distribution involving the left frontal lobe, as depicted in the upper right image of Figure 3. Note also, that there is a small area of hemorrhage underneath the site of the coup impact area on the right which could represent a region of contact surface contusion, but the majority of the hemorrhagic frontal lesions are within the parenchyma, with some extending to the left temporal lobe as well.

**Figure 4:**
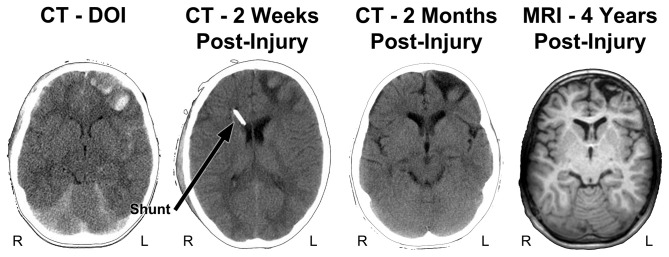
**Parenchymal changes over time from the DOI scan to 4 years post-injury.** The DOI through the scan at 2 months are all from CT and the far right image is a T1-weighted MRI, at 4 years post-injury. In contrast to the CT images shown in Figure 3, the slice level of these images are all at the level of the anterior horn of the lateral ventricle. Note that where the most prominent acute hemorrhages and frontal contusions occur on the DOI scan that this also becomes the center-point for tissue dissolution (encephalomalacia) over time. Shortly after the DOI scan was obtained the child received an intraventricular shunt to aid in intracranial pressure reduction and monitoring. By 2 months post-injury the focal encephalomalacia is very prominent (white arrow) and there is also prominence of the Sylvian fissure (black asterisk) along with ventricular enlargement, indicative of some generalized cerebral volume loss. R, right; L, left.

Viewing these types of focal injuries, the next important consideration to understand is the distribution of biomechanical distortion of the brain that results in this type of damage. Understanding the biomechanics of injury provides clues as to which regions within the brain will receive the greatest shear/strain; hence, where likely pathology may be observed as a result of the greatest deformation of brain parenchyma. Much of the shear/strain pathology occurs at the cellular level, but understanding where and how the macroscopic lesions occur informs where other cellular pathology may reside. Bayly et al. (2012) modeled an occipital impact using a “tagged” MRI protocol and examined deformation strain within the brain. While in the MR scanner, this involved a slight head drop into a sling resulting in ~2–3 *g*, where, g is the acceleration of gravity. This is also depicted in Figure 3. With such minor *g*-forces, no injury occurs and the brain tolerates minimal amounts of movement like this on a regular basis such as with jumping and landing. Nonetheless, the tagged lines which should appear as a rigid grid of intersecting parallel and perpendicular lines, if not deformed, show a wave action throughout the brain with a distinct coup to contrecoup motion. For the actual acceleration/deceleration injury that occurred in the “TBI Case Study” within milliseconds there was rapid and massive movement of the brain within the cranium to produce this lesion pattern which would have put considerable strain across deep white matter (WM) regions including the corpus callosum and especially long coursing fasciculi and subcortical areas, along with the secondary frontal impact. As depicted in Figure 3, because of where the scalp soft-tissue swelling was located, a point of impact was likely on the right posterior lateral aspect of the head with energy translated forward, after the initial impact. Because of the position of the anterior and middle cranial fossa, with this kind of impact there will likely be deformation against boney ridges (Bigler, 2007). As will be shown with MRI studies, depicted in subsequent illustrations, this kind of coup-contrecoup pattern of injury results in multiple isolated as well as dispersed lesions more than just the hemorrhagic shear injuries within the frontal lobe readily viewed in Figure 3. Indeed, part of the reason for this child having lost consciousness with persistent low GCS for several days is the likely disruption of upper brainstem WM tracts that involve the reticular activating as well as diffuse thalamic projection system (Jang et al., 2015), along with the generalized cerebral edema, for which this child underwent shunt placement for better management of intracranial pressure (see Figure 4). Significant strain effects will not necessarily produce classic shear lesions, but may nonetheless induce cytoskeletal changes or alter synaptic integrity that may render inefficiencies in neural conduction. This combined with inflammatory changes result in complex cellular and metabolic pathologies occurring throughout the injured brain. As compelling as the CT images are that a major aspect of trauma-related pathology has been captured, as will be shown the coarse abnormalities in Figure 3 are just the beginning. Remember, the premise in this review is that lesion evolution in TBI is dynamic and especially in the first weeks to months post-injury, ever changing. Therefore, whatever neuroimaging feature is captured at a given time point is but a snapshot at that point in time post-injury.

There is another element associated with the biomechanics of brain injury also revealed by the tagged lines in the model shown in Figure 3 which shows that the movement is never uniform and varies across different brain regions and sites depending on the moment (within milliseconds) from the point of impact to when parenchymal distortion occurs. As has been shown by finite modeling (Kraft et al., 2012), axons are therefore not uniformly affected within a strain field. So depending on the impact energy translated to the brain, the unique and individualized conformity of the brain within the cranial vault, the rotation and distribution of energy forces and brain displacement, some axons may be injured, others not affected. Just these factors alone create heterogeneity so that no two brain injuries are ever identical.

Figure 4 also depicts follow-up CT imaging within the first 2 months of injury along with a T1 weighted MR image at a generally similar level obtained 4 years post-injury. As reflected in the initial images not only are the prominent frontal hemorrhages visible but the brain is swollen, there is subarachnoid blood and loss of sulcal definition throughout the brain and an initial decrease in ventricular size because of generalized cerebral edema. Taken together these initial findings are consistent with diffuse, generalized swelling of the brain. The shunt catheter is viewed in the right anterior horn of the lateral ventricle, used to assist in managing intracranial pressure at the 2-week post-injury scan. The inflammatory aspect of brain trauma represents another pathological feature of the brain injury especially influential at the cellular level in terms of metabolism and blood flow, often considered as a secondary cause of damage following the initial brain injury (Bramlett and Dietrich, 2015). While this review will remain focused on just the structural side of neuroimaging, there are numerous neuroimaging methods including MR spectroscopy, MR-based blood flow measures along with positron emission tomography (PET) and single photon emission computed tomography (SPECT) imaging that can be used in studying TBI (Amyot et al., 2015; Irimia and Van Horn, 2015; Sundman et al., 2015; Wilde et al., 2015; Currie et al., 2016). These later techniques provide more direct information about actual brain activity and metabolic functioning, which is not reflected in just a structural MR scan (Irimia and Van Horn, 2015; Wilde et al., 2015; Koerte et al., 2016). It is beyond the scope of this review to go into any detail of these techniques in reference to neuropsychological outcome studies, but the reader is directed to an entire issue of Neuropsychology Review edited by Sullivan and Bigler (2015) that summarizes the utility of these measures in neurocognitive and neurobehavioral studies.

Beyond the initial impact dynamics and focal pathologies that occur on the DOI, as a consequence of the generalized cerebral edema secondary injury and brain insult occurs (Yu and Kobeissy, 2015). Accordingly, some non-specific cerebral damage evolves over time that is a consequence of the primary and secondary effects of the trauma. As shown in Figure 4 these changes over time can be assessed with structural imaging, often reflected as changes in brain volume. Viewing the DOI CT findings in Figure 4, the anterior horns of the lateral ventricular system have a rather slit-like appearance, probably a reflection of compression from generalized cerebral edema when initially scanned. However, at all follow-up time periods the ventricular system is larger, which can be quantified by measuring what is referred to as the ventricle-to-brain ratio or VBR. VBR can be used to measure how much the ventricular system changes with edema or can be used as a global index of cerebral atrophy (Bigler et al., 2004). When the VBR was done at 4 years post-injury, this child’s VBR was elevated in excess of two standard deviations compared to a normative sample, signifying generalized volume loss.

## What Lesion to Measure?

As shown in Figure 4, within 2 weeks significant parenchymal changes appear in the form of tissue degradation from the DOI scan, reflecting the dynamic features of what was the original traumatic injury. As already stated, quantitatively there is significant loss of parenchymal volume that can be measured as well as viewed. Since CT imaging is based on tissue density, the darker appearance in the 2-week and 2-month follow-up CT scans, where prominent hemorrhagic lesions in the left frontal lobe were originally identifiable on the DOI CT scan (bright white within brain parenchyma in Figures 3, 4), reflects loss of tissue density that evolved over time. This signifies that tissue dissolution along with absorption of much of the extravasated blood has occurred, all-the-while intracranial pressure is going down and the child is coming out of coma and improving cognitively. These observations underscore the dynamic changes and wide variability that occurs when trying to pin down and measure a particular injury-related pathology. Since major aspects of the changes visualized at 2 months become the chronic structural abnormalities viewed in the MR scan at 4 years post-injury, extremely complex histopathological, phagocytic, neuroinflammatory, vascular and structural changes are dynamically occurring in a relatively short period of time.

So when does one establish what is the lesion in TBI and when and how should it be measured? As shown in Figure 4 at 2 months post-injury a distinct region of frontal encephalomalacia has emerged (arrow in upper right image of the CT at 2 months post-injury), with what appears to be a very distinct boundary, by CT standards. Image analysis tools are readily available that could outline the boundaries of encephalomalacia and by knowing the thickness of the CT image and the distance between each scan slice, a volume estimate of the damage could be calculated. Since on CT regions of encephalomalacia reflect CSF prominence one can be certain that pathological changes in brain parenchyma would be captured by performing such an analysis. But is a clearly defined CT region of identifiable encephalomalacia in TBI the sole lesion and the best method for characterizing the damage?

**Figure 5:**
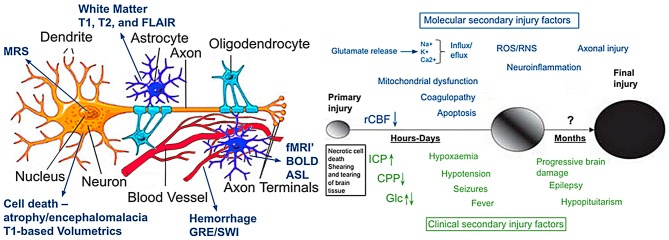
**The illustration on the left is modified from Marklund and Hillered (2011) and Ling et al. (2015) and depicts a typical CNS neuron with supporting cells and blood supply (used with permission).** Although an over simplification, also outlined in the illustration is how different imaging techniques tap different components of cellular or vascular integrity. The image to the right is from Gandy et al. (2014) which they used to portray the molecular pathogenesis of TBI and CTE. It illustrates current concepts of mechanisms underlying primary and secondary injury mechanisms in TBI and how they change overtime. At early times after injury, glutamate release and ionic disturbances (Na^+^, Ca^+^ and K^+^) disrupt energy metabolism and cause other metabolic disturbances that lead to decreases in cerebral blood flow. Mitochondrial dysfunction causes increases in reactive oxygen (ROS) and nitrogen species (RNS) that can cause further cellular injury. Tissue damage evokes neuro-inflammatory changes that emerge later. Injury may be exacerbated by secondary clinical factors including hypoxemia, hypotension, fever and seizures. These secondary molecular and clinical factors lead to progressive tissue damage. Abbreviations: Ca^2+^, calcium ions; CPP, cerebral perfusion pressure; Glc, Glucose; ICP, intracranial pressure; K^+^, potassium; Na^+^, sodium; rCBF, regional cerebral blood flow. Used with permission from the British Journal of Pharmacology and Wiley Publishing.

**Figure 6:**
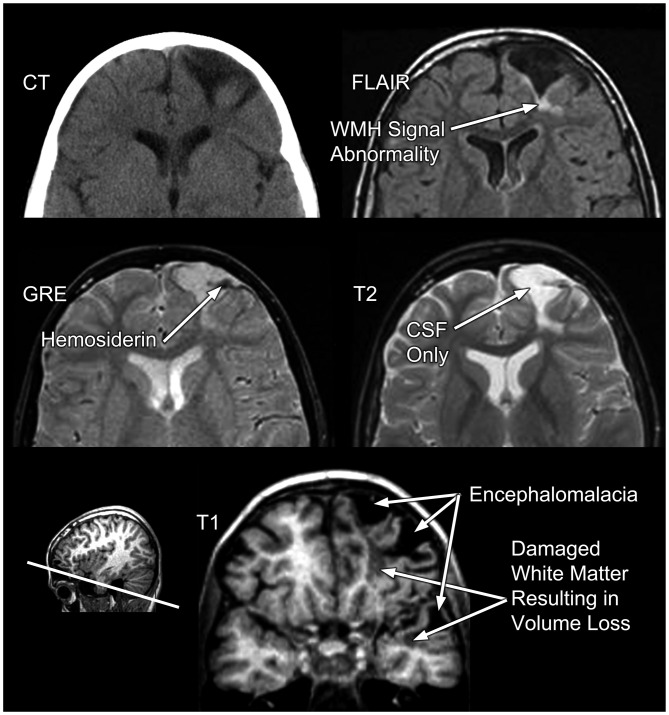
**In the same TBI patient as presented in Figures 3, 4 different MRI sequences yield different facets of the brain injury and residual pathology.** FLAIR, fluid attenuated inversion recovery; MRI sequence GRE, gradient recalled echo MRI sequence. “L” refers to the patient’s left.

**Figure 7:**
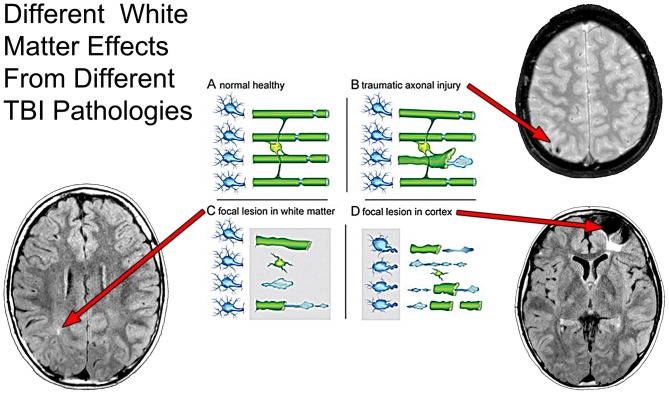
**As taken from Armstrong et al. (2015, 2016) where the effect of axon degeneration on the myelin-oligodendrocyte unit in different pathological scenarios following TBI.** Schematic of a myelin-oligodendrocyte unit in the normal adult brain **(A)** or after different pathologies associated with TBI **(B–D)**. In the healthy condition **(A)**, neurons (blue) in the cerebral cortex (left side of panel) extend axons that project through the WM (right side of panel). Myelin and oligodendrocytes are shown in green. Each oligodendrocyte forms myelin sheaths around multiple axons. Nodes of Ranvier (blue) are specialized regions of axon between adjacent internodal lengths of myelin. Traumatic axonal injury **(B)** causes axon degeneration, as illustrated by a single damaged axon among a set of adjacent intact axons. Axon damage from traumatic axonal injury often initiates at nodes of Ranvier. Damaged axons swell, fragment, and form end bulbs with accumulations of organelles and cytoskeletal elements. Double-layered myelin sheaths often extend out from degenerating axons. The length of these myelin figures exceeds that expected from collapse of the myelin sheath around a degenerating axon. TBI can cause focal lesion areas in WM (**C**, gray box). Focal lesions, for example from microhemorrhages or neuroinflammation, damage a high proportion of axons, oligodendrocytes, and myelin. The cerebral cortex can undergo similar focal tissue destruction (**D**, gray box), particularly in cortical regions underlying the site of an impact to the head. Axons become disconnected from damaged cortical neurons. Degeneration of a high proportion of axons in a WM tract leads to subsequent myelin degradation and oligodendrocyte death. These three scenarios **(B–D)** focus on damage to the neuron and axon to show the relationship to the myelin-oligodendrocyte unit. Scenarios **(C,D)** illustrate loss of myelin as a result of overt tissue damage that includes loss of axons. None of the examples illustrates actual demyelination, i.e., loss of myelin around an intact axon, which can also occur after TBI. Superimposed on this schematic from Armstrong et al. (2015) are different MRI sequences likely sensitive to the different WM pathologies as outlined by Armstrong et al. where a focal WM hyperintensity may reflect focal WM damage (lower left image), whereas a hypointense lesion pattern may indicate TAI (upper right panel) but when there is cortical destruction underlying WM degrades as well, shown by the hyperintense signal just underneath the region of focal encephalomalacia. Used with permission from Elsevier Publishing.

The answer is no, because CT’s dependence on tissue density alone provides just a unidimensional image of brain pathology. Turning to the schematic in Figure 5, too often when TBI is discussed it has been discussed and modeled only as an axonal injury within the context of DAI. However, the basic schematic shown in Figure 5 (adapted in part from Marklund and Hillered, 2011; Gandy et al., 2014; Ling et al., 2015) shows not only the neuron, but glia including the oligodendrocyte derived myelin along with capillaries that are as small and delicate as the neural structures they feed and with which they functionally interact. There is also the extracellular matrix whose role is only becoming known, but equally important and probably involved in the pathological effects of TBI as well (Benarroch, 2015). Parenchymal damage from trauma affects all of these cellular components and fortunately, MRI has a differential capability in detecting different tissue types and related pathology. The CT in this “TBI Case Study” objectivity shows a large area of frontal encephalomalacia but does not show any associated pathology that can readily be separated into more specific components of damage related to WM, gray matter and hemorrhage as well as metabolic or blood flow changes that can be achieved with MRI. As shown in Figure 5 (see also Figures 6, 7), different MR sequences have different capabilities of detecting these different tissue types and pathologies that relate to the source of pathology at the cellular level from TBI. At the macroscopic level, this is shown in Figure 6 which depicts different aspects of trauma related pathology based on different MR sequences, which likely provides unique information about the injury beyond what can be gathered from just CT. Furthermore, a systems biology perspective would suggest that patterns of predominately differing pathology likely have different influences on outcome so they would need to be examined both separately as well as within the overall “system.” Accordingly knowing whether predominate patterns of damage were expressed via changes in WM, gray matter or hemorrhagic pathology or combinations would likely differentially influence outcome.

Taking this latter point further, Figure 7 more specifically depicts how MRI can assist in identifying different microstructural components of the damage from TBI. Using a schematic from Armstrong et al. (2015, 2016) that illustrates how damage from TBI and shear injury may appear at the cellular level, what has been inserted into their schematic is the “TBI Case Study” MR scans that depict different pathologies. In the classic sense of stretch/deformation injury that produces DAI, as previously stated this has been operationalized as centered on the GRE or susceptibility weighted imaging (SWI) MR scan (see Yuh et al., 2013), based on the number of so-called microhemorrhages (hemosiderin foci) identified. As shown in Figures 6, 7B these hypointense (dark) signal abnormalities, also referred to as “microbleeds” show up as punctate small lesions thought to indicate where capillary rupture has occurred from shear injury or as in Figure 6, larger areas where prior contusion has occurred. In theory, if sufficient to shear blood vessels then sufficient to injure axons, with presumed DAI pathology to be present in association with punctate microhemorrhages. In the child featured in the “TBI Case Study” there is also clearly cortical damage, with extensive hyperintense white matter (WMH) signal abnormality observed on the FLAIR MR sequence (see Figures 6, 7D). As a consequence of the cortical damage, associated with deformation, contusion, hemorrhage and edema this WM damage as shown in the schematic may be associated with more widespread pathology and not just selective axonal damage (Figure 7D). Using FLAIR imaging, there is also the punctate WMH signal abnormality that shows up within deep WM as presented in Figure 7C in this child. WMHs have been associated with TBI, especially when they are observed at the border of the gray-white matter junction (Riedy et al., 2016), but they do occur in healthy, asymptomatic individuals with no history of head trauma, and are therefore nonspecific findings (Bigler, 2013). However, in the “TBI Case Study” highlighted in this review, the WMH signal abnormalities likely relate to the trauma sustained from the head injury but their clinical significance has not been fully determined. While GRE/SWI identified microbleeds (hemosiderin deposition) in association with WMHs relate to DAI/TAI as Riedy et al. (2016) have shown, WMHs and SWI-defined trauma-related microbleeds may not overlap (Liu et al., 2016). Therefore, at the systems level detection of abnormalities by these differing MR sequences, they may be tapping different cellular pathologies. Since in Figure 7C, there is no identifiable hemosiderin deposition in the region adjacent to the WMH does this punctate WMH reflect a focal shear lesion or some sort of other focal lesion within the WM? In the Armstrong et al. (2015, 2016) conceptualization a focal WM disruption may reflect underlying axonal pathology that is different than TAI. Where there is more confluent hyperintense signal on FLAIR imaging, this suggests a more extensive degradation of WM integrity which is likely a combination of multiple factors not just shearing (Bigler, 2015). When viewed where the major cortical encephalomalacia has occurred, as depicted in Figure 7D, beneath the large frontal defect, these WM changes likely evolve from multiple pathological factors. Clearly there was cortical destruction, but also from the DOI CT, there were shearing events that occurred within the WM along with mechanical deformation of the cerebral cortex and contusion. So the WM degradation in Figure 7D may be a combination of cortical damage and neuronal loss as well as specific DAI and more general TAI effects within the frontal WM. Indeed, Smitherman et al. (2016) have shown that in terms of coarse prediction of pediatric outcome (Glasgow Outcome Scale-Pediatrics, GOS-P) from TBI total FLAIR-identified WM pathology was the best predictor of global outcome, but not necessarily specific neuropsychological findings.

So are the scan images present in Figure 7, sufficient to capture the “lesion”, or is there more pathology? No, the answer is that more analysis and quantification is needed.

Returning to the T1-weighted image from an oblique coronal slice at the bottom of Figure 6, even at this coarse level there is distinct anatomical definition between the gray cortical ribbon and underlying WM. Furthermore, where the cortical damage has occurred there is visible reduction in the thickness of the cortical mantel that remains (compare the thickness of the right hemisphere cortical ribbon to the more damaged left). Using the T2-weighted images at the same level also helps define boundaries between CSF and what may be parenchyma (refer to Figure 6). Using information from these two sequences permits quantification of overall parenchymal volume loss in this region along with CSF volume increases. This was depicted three-dimensionally in Figure 2 (the left frontal encephalomalacic lesion is depicted in red in the “Organ” level illustration). Also the T1-weighted image can be segmented into white and gray matter and then using an open-source program like FreeSurfer^1^, major ROIs can be identified, parcellated and classified such that volume can be calculated for each ROI (Bigler, 2015). Since the volume of the brain and all of its component parts are age and development dependent by assessing age-specific, healthy, typically-developed individuals a comparison sample can be generated where each ROI is compared to a normative sample. Size relates to function in a positive fashion although from a neuropsychological perspective size/volume correlates typically account for less than a third of the known variance between size/volume and function (Bigler, 2015).

**Figure 8:**
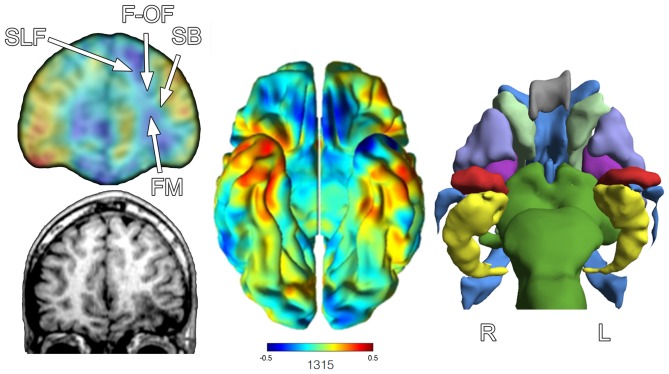
**The image on the left shows at a coronal slice level the Jacobian warping to fit the TBI patient brain to the normative control template.** An actual coronal T1-weighted slice at an approximate level from the anatomical MRI is presented in the lower left. The arrow points to the region of encephalomalacia. The middle image is the full Jacobian analysis depicting the ventral surface of the brain where the degree of “blueness” demonstrates where volume loss has occurred, where oppositely warm colors reflect increase. The image to the right is from FreeSurfer output to generate a 3-D image of various ROIs: red = amygdala, yellow = hippocampus, purple = putamen, gray = corpus callosum, light green = caudate, blue = ventricle, violet = globus pallidus, darker green = brainstem. Note that the left hippocampus, amygdala and putamen are smaller in appearance, empirically confirmed based on FreeSurfer volumetric findings in reference to an age- and demographically-matched control sample. R, right, L, left. White arrows in the upper left image depict general location of four major fasciculi that interface with the frontal lobe. SLF, Superior Longitudinal Fasciculus; F-OF, Fronto-Occipital Fasciculus; SB, Striatal Bundle; FM, Forceps Minor.

From this kind of volumetric approach using automated image analysis techniques, several 100 brain ROIs can be derived, but the individual compilation of such measures becomes cumbersome when attempting to visualize where traumatic changes have occurred. Another approach is to use 3-D techniques that warp the individual brain to a normative template where a Jacobian-modulated extraction of how the patient brain has to be altered to fit the template graphically will display where differences reside in the individual patient compared to the control sample (Avants et al., 2011a,b; Tustison et al., 2014; Khan et al., 2015). Using such an approach if there is substantial loss of parenchymal volume in the patient, the warping to fit the normative standard will depict volume reductions. This is commonly shown in shades of blue that reflect how significant the regional loss is, with the most intense blue representing the greatest volume loss or difference from the normative template. Oppositely, increases are signified by “warm” colors with the greatest increases shown as dark orange to red. Up to this point the “lesion” in this research participant has be highlighted by focal characteristics of signal differences visibly based on the native MR sequence, with the frontal encephalomalacia dominating the coarse visual changes. However, what these Jacobian maps depicted in Figure 8 show not only the expected cortical volume loss from the obvious focal lesions but actually extend to volume differences to regions where there were no visible lesions. For example, in the coronal map on the left side of Figure 8, note that in the right frontal lobe it has less volume as well, even though the T1 anatomical image does not reflect focal damage. When the ventral surface is shown (middle view) the most intense blues are in the left inferior frontal and temporal polar areas, nicely corresponding to where the obvious encephalomalacia has occurred but much of the inferior, orbitofrontal region of the right is also reduced. The visible effects of volume loss within subcortical areas as depicted from the FreeSurfer output as shown in Figure 8 on the right side of the figure, demonstrates visibly smaller left hippocampus, amygdala and putamen, when compared to right hemisphere counterparts as well as to a normative sample. So not only are there “lesions” in the traditional sense of being visibly identifiable as shown in Figures 4, 6, 7, there are widespread volumetric changes that have pathologically altered brain structure.

One explanation for volume loss and changes in shape that occur distal to the focal pathology is the loss in connectivity (Hayes et al., 2016). Also shown in Figure 8 is the relative position of major fasiculi that interface the frontal lobe with the rest of the brain. Because of the focal pathology on the left, WM tracts that would normally interconnect with the inferior frontal lobe on the right via the forceps minor (FM) drop out affecting the volume and shape of structures that were once interconnected.

**Figure 9:**
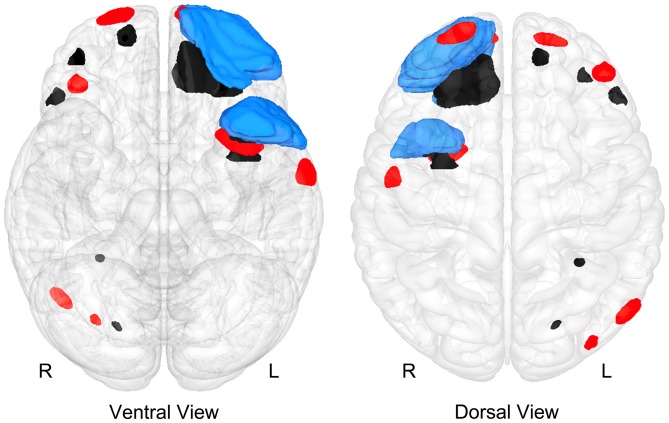
**A “glass-brain” image of the Case Study Child with TBI depicting where focal abnormalities could be isolated as identified by T1 and T2 imaging for encephalomalacia (blue), region of focal hemosiderin deposition (red) and WM hyperintensities (black).** Noteworthy is that while the majority of these focal lesions involve frontal and temporal lobe regions, there is little overlap of the lesions.

As already mentioned, the GRE sequence is particularly sensitive to parenchymal iron deposition and identifying regions of hemosiderin and likely prior hemorrhage (Chappell et al., 1992). Of particular interest from a neuroinflammatory perspective is that presence of hemosiderin may relate to localized neuroinflammation (Schrag et al., 2010; Logsdon et al., 2015). Therefore, where GRE or SWI—the SWI sequence is superior to the standard GRE—findings detect prior hemorrhages from TBI may serve a dual role: (1) demonstrates the location of prominent shearing; and (2) identify locations potentially more susceptible to inflammatory response because they indicate where focal injury has occurred. Figure 9 also includes the locations of microbleed and focal areas of hemosiderin deposition, which as visualized in this illustration may be independent of where either WM pathology or focal atrophy have occurred. This would argue that GRE/SWI abnormalities should be analyzed separately and any study that examines just one of these MR sequences will not be addressing all of the different ways in which the brain may be damaged and that damage quantified.

Does the 3-D approach presented in Figures 2, 8, 9 now capture the totality of the “lesions” associated with TBI in this case study? No.

Degeneration that occurs distal to the site of axonal injury is referred to as Wallerian degeneration, but the role that such degeneration plays in TBI is not fully understood (Maxwell, 2015). Neuronal health and integrity is dependent on cellular interaction and when a single neuron within a network of neurons is injured, loss of trophic input to subsequent neurons in the chain will either disrupt the network, requiring network adjustment and work-around to maintain function, or the potential for downstream neuronal cell death due to lack of input (Nave, 2010; Conforti et al., 2014). What this means from a “systems” perspective is that the neuroimaging identified lesion does not occur in isolation, with potential influences very distal to where the lesion may be. As a consequence of variations of Wallerian effects, volume loss, structural changes in shape and contour may occur distal to wherever a more focal pathology may be identified, as explained above and shown in Figure 8; therefore, measuring just where the focal pathology is located may be misleading and incomplete in describing the totality of the effects of a focal lesion.

For example, the Jacobian-warped image of this TBI child’s brain in Figure 8 (upper left image) shows volume reduction in multiple regions of the right frontal lobe that did not sustain the kind of focal damage that occurred on the left. Also of interest, note that there are also regions of volume increase as reflected in areas of red-orange (middle ventral view of Figure 8). Specifically, the temporal polar area of the right is larger than the age-appropriate pediatric template from control children without brain injury. There are no identifiable lesions in the right temporal area, but the left was injured. Since the MRI was obtained 4 years post-injury, does this represent regions of developmental compensation in the less affected right temporal lobe? Does hypertrophy in areas not or less injured reflect mechanisms of recovery? Adaptation? Not known, but an interesting speculation. What can be said quantitatively though, is rather startling. Based on the FreeSurfer lesion quantification, total lesion volume loss in this child (defined as the total of CSF in regions of focal pathology, total WM hyperintense signal and abnormal GRE findings) is close to a 100 cc, a volume loss at about 9% of total brain volume. If one now takes a conservative estimate of ~80,000 neurons with 4.5 million synapses per 1 mm of neural tissue (see Insel and Landis, 2013), the observable damage within these different illustrations would reflect billions of damaged or lost cells and trillions or more of disrupted or lost synaptic connections.

Despite the various objective abnormalities that these neuroimaging studies demonstrate, by GOS-P standards the child featured in these analyses has had a good outcome. As previously mentioned testing at age 12 revealed an above average FSIQ of 109. This child was also adequately functioning within a regular public school classroom placement. Given the ROI volume loss and numerous focal lesions, a conundrum emerges as to what significance to give to the abnormalities or should this be discussed in terms of potential compensatory changes and resiliency that have occurred over the 4 years since being injured? From a “systems biology” perspective, living systems do so through adaptation, so what neuroimaging shows should not just be where pathology may reside, but also how the system adapts.

From the above discussion and what has been illustrated in Figures 1–8, neuroimaging provides excellent methods for representing TBI-related pathology at the tissue and organ level, essential from a systems biology perspective as presented in Figure 2. The question of what is the lesion, how measured and expressed clinically and in research has still not been answered because part of that definition will be how pathology at the tissue and organ (brain) level affects neurobehavioral functioning at a systemic level. As such, the next “systems” level discussion turns to one of neural networks and neuroimaging, in an attempt to address the bottom rung of the systems pyramid as depicted in Figure 2.

## Systemic Level in Health and Dysfunction: Brain Imaging of Networks

The lesions and structural abnormalities as shown so far can be measured in MR signal intensity, volume, surface area, contour, thickness of a cortical region and their location all accurately quantified and depicted in 3-D. However, if an identifiable but abnormal signal from a particular MR sequence constitutes a “lesion” not detectable by another MR sequence means that how a “lesion” is defined and measured depends on the sequence and what is being measured. Thus, integration of all neuroimaging information needs to include all MR sequences, not just limiting consideration of information from one. As shown in Figure 9 the locus and distribution of focal abnormalities is entirely dependent on the MR sequence where much of these different abnormalities do not overlap in the “TBI Case Study” presented in this review.

Furthermore, the neuroimaging presentation thus far has focused on just the structural side of neuroimaging and lesion identification, but not on the potential consequences of how lesions disrupt networks, from small local networks, to large integrated networks regulating behavior, cognition and emotion. The traditional size, volume and location of trauma-related abnormalities are just part of the story. Probably even more important at the systemic level, is how a lesion affects the neural network (Catani and Thiebaut de Schotten, 2008). Fortunately, there are numerous neuroimaging techniques that provide methods for defining networks and assessing their integrity (Sorg et al., 2015).

**Figure 10:**
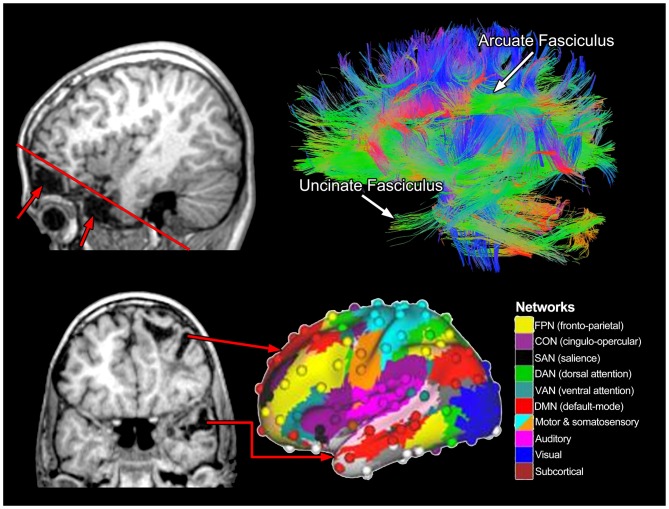
**The upper right image represents a lateral view of whole-brain tractography from diffusion tensor imaging (DTI) where two major fasciculi are identified.** The color schema reflects fiber tract orientation where green reveals anterior-posterior coursing tracts, blue vertically oriented and warm colors (orange-red), reflect laterally oriented tracts. The upper left sagittal view shows the inferior frontal (top arrow) and temporal (bottom arrow) lobe encephalomalacia with the oblique red line showing the angle of the coronal cut as shown in the bottom left T1-weighted MRI. As adapted from Cole et al. (2013) and used with permission from Nature Publishing Group is a representation of various brain networks, color coded by networks identified in the right panel. The red arrows in the lower left panel come from distinct areas of damage that would likely affect various networks associated with frontal and temporal lobe function.

Figure 10 uses the MR technique referred to as diffusion tensor imaging (DTI) to illustrate aggregate WM tracts in the brain of the left hemisphere in a healthy individual. Returning to the structural imaging in the TBI Case Study with severe injury, as impressive as the focal pathology may be, it is not the focal damage, *per se*, that is most disruptive to functional outcome but how focal damage and related pathologies affect pathways and neural networks. In Figure 10, the upper left sagittal T1-weighted MRI depicts the frontal and temporal polar damage with the red line depicting the angle of the cut for the coronal image shown in the bottom left of Figure 10. Viewing the extent of the focal damage in the context of DTI-derived pathways, all of the input and output connections would be affected. Two major pathways shown in Figure 10—the arcuate (part of the superior longitudinal fasciculus [SLF]) and uncinate fasciculi (temporal lobe to orbitofrontal connectivity)—both would be disrupted along with the frontal and temporal distribution of the cingulum bundle (buried and not distinctly visible in Figure 10 (see figure caption), inferior occipitofrontal fasciculus (again buried and not specifically viewable in Figure 10 but courses along the base of the frontal and occipital lobes). These pathways actually participate in multiple networks and should not be viewed, as in earlier days of neuroscience, as being dedicated to a specific function, but to multiple cognitive and behavioral functions.

Beyond the scope of this review, but briefly summarized herein there is a tremendous amount of neuroimaging research on brain networks that involves functional MRI (fMRI) often combined with other electrophysiological measures and techniques like DTI. Figure 10 also presents the cortical network schematic developed by Cole et al. (2013), representative of 10 different networks derived from a combination of these techniques. From this perspective, whatever lesion or pathology may exist from a TBI, the neurobehavioral consequences will be in terms of which networks are damaged, by how much and how adaptive the networks may be after being injured (see Hayes et al., 2016).

**Figure 11:**
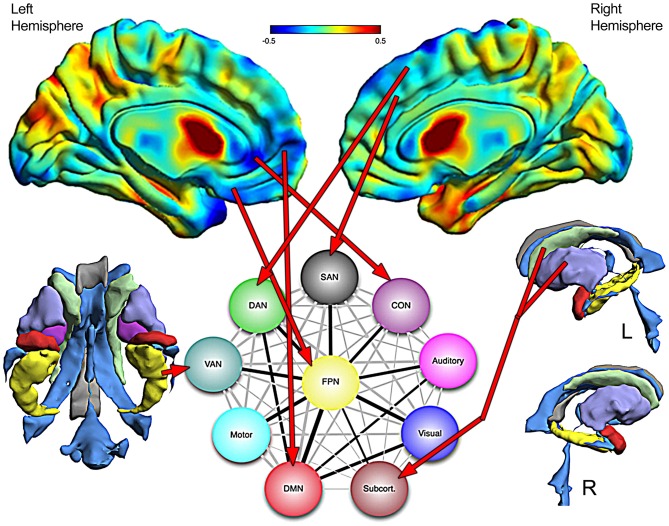
**The top images are the Jacobian warped brain maps that reflect volume loss or increase in relation to a normative template (see Figure 8 for color description).** Assuming that where significant volume reduction has occurred would reflect damage to networks dependent on those areas, a schematic network map from Cole et al. (2013) is used to project where damage is and what networks would be affected. The FreeSurfer derived 3-D images of the ventral view of subcortical areas is shown on the left (without the brainstem—see Figure 8) and lateral views on the right, with red arrows pointing to networks that are likely affected by the damage. Network diagram is used with permission from Nature Publishing Group.

One important feature of the Cole et al. (2013) investigation was the central role of the frontoparietal network (FPN) as an integrative and across-network connectivity center. Figure 11, adapted from Cole et al. (2013) portrays a simple network connectivity map that depicts the central role of the FPN within the overall integrated whole-brain network. In the TBI Case Study by using abnormal FreeSurfer (>2.0 SD’s below an age-matched norm) volumetric ROI findings combined with where the most significant cortical volume losses were identified via Jacobian warping as indicators of residual damage which network affected can be plotted (red arrows in Figure 11). From a systems biology perspective, identifying pathology is just part of the challenge, but how pathology influences the organism at the systemic level (as highlighted in Figure 2) is the most important challenge because therein is the link between damage, neural networks and behavior. In the case of TBI this would be to utilize neuroimaging information about pathology—not just were visible lesions are but where volume loss has occurred as well—and use that information in the examination of cognition, emotion and behavior. How to integrate this with neurobehavioral outcome, to be discussed in the next section, will require a major conceptual shift in how neuropsychology has approached assessment.

## The Limits of Traditional Neuropsychological Assessment

To objectively study human behavior it has to be measured in some fashion, which includes metrics to assess cognitive and emotional functioning. This was a major aspect of Marr’s (1971) original outline for a systems approach related to cognition—the necessity of accurately measuring the target behavior. To achieve the initial goals of characterizing the neurocognitive and neurobehavioral correlates of the brain when damaged, neuropsychology emerged mid-20th century in an era where non-invasive neuroimaging did not exist. Out of necessity, the founders of this field had to be creative in terms of natural observation and capitalized on coarse measures of brain damage, like aphasia, hemiplegia or hemisensory deficits from TBI and other disorders (i.e., stroke, neoplasm) to standardize certain neuropsychological measures (see Bigler, 2009). Using a normative approach with test scores established in age-typical healthy controls, neuropsychological assessment techniques were developed where the influence of brain damage/dysfunction was viewed in terms of discrepant performance from the norm for a particular measure like memory or perceptual processing (Lezak et al., 2012). In the formative years of clinical neuropsychology the interpretation of such findings was viewed from a perspective of lateralization, localization and modular control (Ross and Long, 1990; Ross et al., 1990), but not networks (Catani et al., 2012). Interestingly, in terms of blunt dissection techniques all of the major fasciculi that now can be so eloquently shown *in vivo* with DTI techniques, as presented in Figure 10 were known by early 20th Century. Major speculations about their role in behavior were central to connectionist theories of brain function, championed by Geschwind’s seminal contributions beginning in the 1950’s and extending into the beginnings of neuroimaging, including TBI (Rubens et al., 1977). However, even in Geschwind’s era, the emphasis was more on where a particular lesion could be disrupting a pathway or pathways rather than the multiple ways in which pathology and lesions may be expressed in TBI (Filley, 2010, 2011).

Despite 21st Century technology and the technological feats of modern neuroimaging, the basic “tools” of neuropsychological assessment still use a “paper-and-pencil” foundation introduced by Alfred Binet in the early 1900’s (Binet and Simon, 1905). In most cases if a task is timed, it is measured in seconds to minutes with the use of a hand-held stop watch, whereas neural processing occurs in millisecond timeframes. For many disorders this traditional neuropsychological approach works well, just like the current reflex hammer and stethoscope that came into general use about the same time and have required no digital update. However, in TBI, as shown in this review, abnormalities are dynamic, ever changing and affecting the brain in unique ways where at each time point post-injury there is a mixture of pathology in conjunction with adaption and recovery. Is the neuropsychological task measuring a deficit, adaptation or an unaffected function? However, an even bigger problem for conventional neuropsychological assessment is that except for certain aspects of motor, sensory and some language tests, standardized traditional assessment methods have *not* been designed to specifically assess network integrity as outlined in Figure 10. Most traditional assessment tools do not tap singular networks, but a multiplicity of them working in concert. Furthermore, neuropsychological test scores are typically reported as aggregate scores reflective of an operationally defined construct in reference to a normative standard not specifically tied to a particular brain variable. For example, in the TBI Case Study presented herein what is the relevance of the FSIQ score of 109 in reflecting how and where the brain has been damaged along with what neural systems are affected without knowing information gathered from neuroimaging? As already presented the neuroimaging in this child shows extensive damage, so what information does an IQ metric convey about network damage and the underlying pathology sustained by this child? The answer is that by itself and without neuroimaging findings factored in, not much.

To further illustrate this point, a speed of processing metric will be used. Processing speed deficits represent a common finding in those with TBI, particularly related to the diffuseness of injury and amount of WM damage (Lezak et al., 2012). In the TBI Case Study the child’s PSI on the WISC-IV was 94, which is a standard deviation (15 points) below the FSIQ score. While the PSI score remains in the low average range, being one standard deviation below overall IQ would be consistent with reduced processing speed as a consequence of the severe TBI. From the pathology shown in Figure 11, such a result is not surprising given the frontal damage, the fact that there is reduced volume in the anterior corpus callosum and cingulate as shown by the Jacobian warping, along with quantitative analyses that show more than a standard deviation reduction in overall WM volume. But which of these regions and precisely which major brain fasciculi are affected cannot be ascertained with a traditional neuropsychological approach like this. All that can be concluded is that processing speed is reduced, but without further integration of both structural and functional neuroimaging whether this is universally reduced processing speed or regional cannot be determined. What is needed is a systems approach where functional techniques like fMRI and/or electrophysiological procedures are incorporated into a multimodal structural MR that more specifically assesses pathway integrity related to function and directly measure specific network efficiency and processing speed. For example, the current review concentrates mostly on anatomical features of structural imaging where anatomical findings do not necessarily reveal function; but fMRI and electrophysiological techniques reveal patterns of activation and neural engagement measured with millisecond precision directly extracted from brain activation patterns. From a systems perspective if these different levels of structural and functional neuroimaging could be integrated it should lead to an improved understanding as to how the damaged brain is functioning.

If the neuropsychological assessment tool is to be integrated into a multimodal assessment technique, this also means abandoning 100-year old traditional methods. For example, the PSI task used with this child measures a myriad of cognitive functions, not how they are interconnected or processing time between them. Returning to Figures 10 and 11, processing speed indices need to be developed more specifically that tap within and between networks as well as those that assess overall network integration and integrity and do it in real time brain processing speed, which is in milliseconds. Cognitive neuroscience paradigms for assessing processing speed attempt to be more domain and network specific and therefore tend to use reaction time and/or some event-related electrophysiological or fMRI blood oxygen level dependent (BOLD) activation tasks (Dockree and Robertson, 2011; Nilsson et al., 2014). Using the above mentioned techniques combined with MR-based functional connectivity (fc) mapping the potential for clinical application is being realized (McAndrews, 2014). This work includes having the patient not even engaged in a cognitive task, but using resting state (rs) fc mapping techniques to derive brain networks (Barkhof et al., 2014; Tracy and Doucet, 2015). These rs-fcMRI studies have not found their way into broad clinical application at this point, but that is just a matter of time before there is a more complete integration across cognitive neuroscience, clinical neuropsychology and functional neuroimaging to the point cognition and behavior are entirely assessed within the domain of brain imaging. The next step, within a systems biology framework would be to concomitantly develop such measures specific to the TBI patient and track how neural systems and networks either come back online, adapt or remain impaired. The next section provides some thoughts on how this might be accomplished.

## Systems Biology and Neuropsychology

This review began with the very basic model offered by Vodovotz and An (2015) as to what should constitute a translational systems biology approach to studying a disorder. As presented in this review contemporary and advanced neuroimaging methods certainly provide critical information at the tissue, organ and systemic level. But there remain major gaps, with a most prominent one being how to measure cognition and neurobehavioral outcome and link such findings to neuroimaging variables.

Lisman (2015) reviews 21st Century challenges for understanding the brain as it relates to cognition using the systems perspective introduced by Marr (1971) which emphasized a neural network approach. As previously stated, central to Marr’s (1971) systems approach is defining how behavior and cognition can be measured. Expanding on the foundation laid by Marr (1971), Lisman outlines the following systems framework to better understand cognition: “First, the functional properties of the process must be defined and behaviorally characterized. Next, the computational algorithm that performs that process must be identified. Finally, how neurons and their network connections lead to the execution of that algorithm must be determined (p. 864)”. Juxtaposed to these statements are those of Cipolotti and Warrington (1995) on the neuropsychological assumption that “brain damage can selectively disrupt some components of a cognitive system (p. 655)” and thereby influence test performance which in turn acts as a marker of impaired brain function.

As discussed in the previous section, traditional neuropsychological techniques mostly tap global functions. For a neuropsychological test to be useful in specifically assessing a TBI patient, it must reliably assess the cognitive/behavioral dimension it was purportedly designed to assess and equally important, exhibit decrements in performance levels when those neural systems are damaged. Most traditional test methods in neuropsychology have been developed based on presumed domains of cognitive function, like memory or executive ability, respectively, often thought to reflect “frontal Lobe” and “temporal lobe” integrity. However, using executive functioning as an example of what is wrong with current use of neuropsychological measures in TBI, as shown in Figure 10, there are regions within the frontal, parietal and temporal lobe and their connectivity that make up the executive network. Taking the TBI Case Study presented in this review, what is not known is how do the different lesions and abnormalities as shown in Figures 8–10 influence test performance. Is test performance more influenced by the WM pathology, the focal encephalomalacia, the loci of hemorrhagic lesions or the regional and whole brain degenerative changes that occurred or the integration of all of these pathologies?

Neuroimaging has a distinct role to play in how to best characterize and quantify “brain damage” and relate such changes in neuropsychological test performance but the field has only recently begun to address these issues (Irimia and Van Horn, 2015). Tradition is hard to break but old tests developed during a non-digital era are likely not the answer. New assessment techniques aided by advances associated with computer-based assessments, especially those using virtual techniques (Parsons, 2015) will likely be capable of better defining “the functional properties of the process” as pointed out by Lisman. Integrated with functional neuroimaging and electrophysiological measures, neuroimaging methods could also be instrumental in developing “computational algorithms” to more fully explain the workings of these neural systems that guide cognition and behavior. Interestingly, algorithms applied to modeling processing speed within networks already have been introduced (van der Helm, 2012). Similarly neuroimaging algorithms that improve detection of WM pathology related to slowed processing predictive of cognitive impairment are being developed (Jokinen et al., 2015).

However, to fully achieve the “computational algorithms” that addresses how “neurons and their network connections” lead to cognition and behavior will require animal models. Fortunately, such translational approaches are being developed with similar structural and fMRI and related neuroimaging techniques used with both animals and humans to study the effects of TBI (Kim et al., 2014; Gozzi and Schwarz, 2015; Meabon et al., 2016).

Another criticism with traditional neuropsychological assessment has been that it is overly focused on assessing cognition, especially within a strictly controlled environment, such as a laboratory setting. Unfortunately, as Parsons (2015) points out lab-based neuropsychological assessment does not necessarily mimic real-world circumstances where lots of competing sensory stimuli, intra-individual variables and environmental conditions may be present. The potential to overcome some of these limitations may be accomplished with virtual assessments within a neuroimaging environment, especially if those assessments were expanded to include emotional stimuli and their influence on standard measures of memory, executive, language and visual-spatial functions. Traditional neuropsychological assessment often ignores or incompletely assesses the emotional state of the patient, or when it is evaluated, is done purely by the patient completing a questionnaire. Likewise, processing speed is currently measured as a separate metric, not part of each domain being assessed and certainly not in the context of how fluctuations in emotion may affect cognition. All of this is probably limiting our understanding of how brain, mind and cognition function normally as well as in those with TBI (see Cromwell and Panksepp, 2011).

## Data Presentation and Conclusions

**Figure 12:**
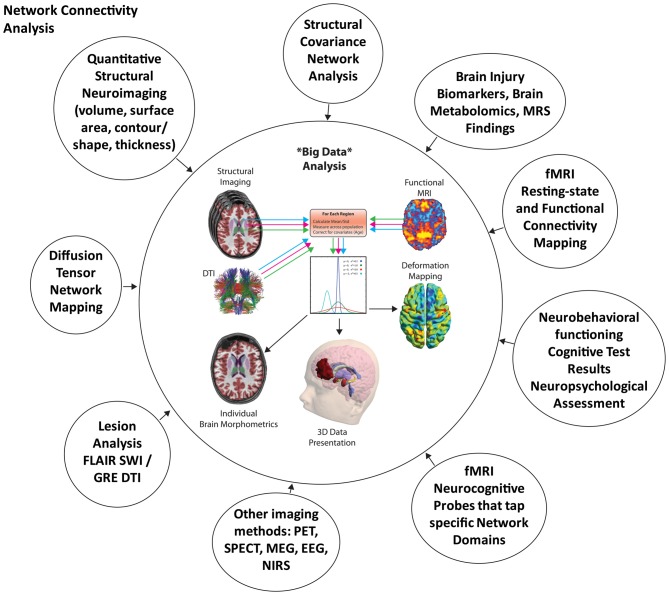
**Using neuroimaging findings to better understand the effects of TBI within a systems biology approach, the various circles highlight different neuroimaging and assessment methods that tap different levels of systems relations and/or pathology that could contribute to an integrative analysis of outcome from brain injury.** At this point, this is merely a schematic representation showing how normative databases could be generated from different neuroimaging methods that yield unique information. For example, the different types of scans and scan sequences can be mined for structural, DTI and functional imaging metrics so that individual comparisons can be made within each data point in reference to a normative sample (center boxes reflecting statistical comparisons to normative samples), with the output displayed on a 3-D image of the brain. For this illustration just the structural defect in the left frontal region is highlighted in the 3-D image at the bottom. The colored lines represent different metrics extracted from the scan such that a statistical comparison can be made between a normative imaging dataset where individual patient neuroimaging data can be contrasted. Ultimately using neurocognitive probes based on some activation technique from functional neuroimaging integrated with correlative techniques that examine neuropsychological test findings and network integrity results, such findings could be presented in terms of their clinical relevance by a color-coded method that highlights where the most robust relations are.

As reviewed to this point, there are elegant neuroimaging methods differentially sensitive to trauma-related pathologies associated with TBI and likewise there are excellent methods to quantify these abnormalities but how should they be shown and integrated with neuropsychological findings? In the very beginning of neuroimaging, scan findings used in neuropsychological outcome studies of TBI involved simple and typically singular metrics like presence/absence of an abnormality on CT or a global measure of brain atrophy (Cullum and Bigler, 1986; Levin et al., 1987). With 21st Century advances, how to handle “Big Data” is now at the forefront with the neuroimaging field likely pulling from genomic, proteomic and other large scale research endeavors to generate the best methods for image display (Toga et al., 2015; Das et al., 2016). As shown in Figure 12 a wealth of image analysis tools exist that can be applied to the quantification of structural and functional effects of TBI, but there is no agreed upon method to integrate these data into a meaningful presentation that capitalizes on all of the available information. From the automated to semi-automated methods now available for image analysis, a single case like that used in this article could involve thousands of data points, just from the perspective of the structural neuroimaging findings whereas if functional neuroimaging were added tens of thousands of data points could be part of the algorithm. The end product for the patient, however, should be something that distills all of this information into something that is relevant to outcome and straightforward to interpret.

Neuropsychology as currently applied can only infer that a particular low score actually reflects an impairment but neuropsychological test findings are not capable of specifying which neural structure(s) or systems are specifically affected without neuroimaging. This needs to be the first step of integration. From a systems biology approach this begins at the tissue level and below (see Figure 2). As of this writing DTI and MRS techniques are the only ones considered to more directly tap the “micro” environment of the brain. Accordingly, for the TBI patient, the first level of analysis may be something that assesses WM patency and general metabolic integrity of the brain. If TBI is selectively disruptive to WM then may be the analysis begins with these MRI methods that examine basic microstructure and metabolic integrity. Both of these techniques also provide some metrics that potentially address neuroinflammation and neuroinflammatory changes over time. Next, volume, thickness, shape and contour of a structure have general implications for parenchymal integrity at the organ level. SWI and FLAIR sequence findings have specific relevance within the domain of traumatic lesions, where lesion localization, size and type likely also relate to findings at the tissue and organ level (Kuceyeski et al., 2015). It may be that the proximity to a focal lesion is an important variable that alters regional tissue shape, size and contour. There are metrics within each approach that can be compared to age, sex and demographic specific normative samples, which could then be used to define where neuroanatomical and neuropathological differences exist in the individual who has sustained a TBI. Combining DTI and other morphometric analyses with rs-fcMRI mapping could approach network analyses and connectivity mapping, showing strength and weaknesses of frank abnormalities within a network in the TBI patient. The end point of such analyses would address the tissue and organ level of brain structure and ultimately shown in some 3-D format an easily visualized image of where pathology resides and its relation to outcome, as has been presented for the case study (see Figures 8, 9, 11, 12).

Next, to understand function at the systemic level using neurocognitive and neurobehavioral test results along with functional activation imaging findings, these tissue and organ level data points would be statistically assessed in relation to the neurocognitive and neurobehavioral test results using machine learning and probabilistic frameworks for classification and differentiation. Features of such an approach have been undertaken for degenerative diseases (Klöppel et al., 2012), neuropsychiatric disorders (Wu et al., 2016), stroke (Kuceyeski et al., 2015) and even TBI (Mitra et al., 2016). At this time all of these studies are mostly demonstrations of “proof of concept” as there is no uniform or agreed upon standard as how these complex analyses should be done and data displayed.

While the above outline is overly broad, it does attempt to take a systems biology approach starting at a tissue level of analysis moving to whole brain and neuropsychological integration. Such an ambitious endeavor will require large sample sizes to begin the process of extracting the most meaningful neuroimaging variables that relate to neurocognitive and neurobehavioral outcome in TBI. Such efforts have actually begun (Mirzaalian et al., 2016; Wilde et al., in press). Despite these obstacles, using such a perspective, should yield novel insights in how best to extract the most meaningful and predicative information from a scan. In that sense, this review returns to Figure 2 where the most important presentation of data may come via which level in the system is being addressed and how that level has been affected, adapted or returned to baseline. The current challenge to the TBI investigator and clinician is how to best bring these rich data gathering methods together to better understand TBI and help improve diagnosis, treatment and recovery.

## Author Contributions

The author confirms being the sole contributor of this work and approved it for publication.

## Conflict of Interest Statement

EDB co-directs the Neuropsychological Assessment and Research Laboratory at Brigham Young University which provides forensic consultation. The reviewer DKC and handling Editor declared their shared affiliation, and the handling Editor states that the process nevertheless met the standards of a fair and objective review.

## Footnotes

^1^http://freesurfer.net
